# Cultural psychiatry in Flanders, Belgium: an exploratory focus group study and position statement

**DOI:** 10.1192/j.eurpsy.2026.10165

**Published:** 2026-02-13

**Authors:** Ruben Willems, Niels Albert, Kris Van den Broeck, Peter Niemegeers, Maarten Van Den Bossche, Sana Sadat Mohammad Rafael Nazari, Lukas Claus, Seline Van den Ameele, Johan Detraux, Marc De Hert, Meryam Scholar-Ocak, Geert Dom, Stefaan Pleysier, Kirsten Catthoor

**Affiliations:** 1 Interuniversity Center of Health Economic Research (I-CHER), Department of Public Health and Primary Care, Ghent University, Ghent, Belgium; 2 Flemish Association of Psychiatry, Kortenberg, Belgium; 3 Faculty of Medicine and Health Sciences, University of Antwerp, Antwerp, Belgium; 4 Family Medicine and Population Health (FAMPOP), University of Antwerp, Antwerp, Belgium; 5 Psyche, Belgium; 6 Collaborative Antwerp Psychiatric Research Institute (CAPRI), University of Antwerp, Antwerp, Belgium; 7 PZ Stuivenberg, Ziekenhuis aan de Stroom, Antwerp, Belgium; 8 Geriatric Psychiatry, University Psychiatric Center KU Leuven, KU Leuven, Leuven, Belgium; 9 Neuropsychiatry, Department of Neuroscience, Leuven Brain Institute, KU Leuven, Leuven, Belgium; 10 Vrije Universiteit Brussel, Brussels, Belgium; 11 Expert by Experience, Belgium; 12 Department of Psychiatry and Medical Psychology, Brugmann University Hospital, Brussels, Belgium; 13 University Psychiatric Center KU Leuven, KU Leuven, Leuven, Belgium; 14 Antwerp Health Law and Health Ethics Chair, University of Antwerp, Antwerp, Belgium; 15 Charité University Hospital Berlin, Charité Universitatsmedicin Berlin, Germany; 16 Multiversum Zorggroep, Boechout, Belgium

**Keywords:** cultural psychiatry, mental health, migrants, policy, qualitative research

## Abstract

**Background:**

As in other European countries, mental healthcare in Belgium has to deal with the increasing cultural diversity that exists within society. However, commitment of the Belgian healthcare system toward cultural diversity remains weak, and clear guidelines on culturally competent psychiatric practice are still lacking.

**Methods:**

Three focus groups with professional caregivers, three with adult patients, and one with young adults in the transition age were organized. The seven focus groups each consisted of 5–10 participants. Two brainstorming sessions with a total of 15 experts were organized a priori to delineate focus group topics. Data analysis software MAXQDA 24 was used for thematic analysis.

**Results:**

The thematic tree consists of the central theme “culturally sensitive mental healthcare” with five main themes (i.e., vulnerable population, language barrier, mental healthcare stigma, spirituality/religion, Western vs non-Western frame of reference). These themes are further stratified into a number of subthemes and one overarching theme (i.e., diversity policy). The themes have resulted in six recommendations to improve cultural psychiatric care. These recommendations underscore the vulnerability of the target patient population, specific training needs, the need for professional interpreters and intercultural mediators, the place of religion and spirituality in therapy, reflexivity as core competence, and the need to establish reference centers.

**Conclusions:**

The six recommendations provide a scientifically sound base to develop focused and effective mental health policies at the governmental, organizational, and patient level. Continued attention to the importance of cultural sensitivity in mental healthcare provision remains important, particularly in countries that are lagging behind.

## Introduction

Migrants and refugees often face significant barriers in access to and quality of care [1]. These disparities are considered to result from individual, provider, and system-level factors, including stigmatization, language and communication barriers, cultural differences, administrative and legal obstacles, insurance issues, and both intentional and unintentional discrimination [1, 2].

Transcultural psychiatry emerged as a field to address the challenges of providing psychiatric care across cultures. It addresses inequities so that every patient can receive the same quality of care [3]. Numerous psychiatric organizations developed programs and guidelines to support these patient populations [4, 5]. The European Psychiatric Association (EPA), for instance, developed the EPA guidance on mental healthcare of migrants [5], with an update of the latter in current preparation. Over time, transcultural psychiatry has evolved into cultural psychiatry. Cultural psychiatry acknowledges that all psychiatry is culturally embedded, moving beyond “our versus other culture” dichotomies. It examines how culture shapes mental health experiences, diagnosis, and treatment [6]. Recent papers have focused on the role of multi-level structural social inequities (e.g., policy, institutional racism, education, employment) [7–9] and interacting identity layers (e.g., religion, ethnicity, gender, socioeconomic status) [10–12] in shaping mental health problems and mental health-seeking behavior. Decolonial psychiatry, a movement challenging Western-centric mental health models, has increasingly received attention as well [7, 13–15].

As in other European countries, the society gets culturally more diverse, and appropriate care for these culturally diverse groups is a priority [16]. Its colonial past, long history of labour migration, and strategic position in the European Union have contributed to this diversity [17]. Around 20% of the people with a Belgian nationality have a migrant background (16.4% in Flanders); 13.7% hold another nationality (11% in Flanders), with the largest non-European group from North Africa [18]. Until today, Belgium has addressed ethnic mental health inequalities only to a limited extent. People with a Moroccan or Turkish background in Belgium face significant and growing mental health disparities compared to non-migrant Belgians [19]. A 2019 report of the Belgian Health Care Knowledge Center [20], as well as a 2022 narrative review [21], highlighted several shortcomings in Belgian mental healthcare for asylum seekers. Moreover, to the best of our knowledge, no clear Belgian guidelines exist on how to carefully and ethically engage with minority groups, including refugees, asylum seekers, and other non-Western migrants in mental healthcare. Guidance is lacking on ensuring smooth access to care, appropriate diagnostic and treatment approaches, family involvement, and integration of a person’s cultural background. The Flemish Association of Psychiatry (FAP) has identified this gap as highly problematic. Based on an exploratory focus group study with clinicians and non-Western migrant patients, this paper aims to formulate policy recommendations to strengthen cultural psychiatry in Flanders, Belgium.

## Methods

### Context

Belgium is a Western European high-income country that enjoys commendable health system performance across various indicators such as healthcare rights and population satisfaction with the availability of quality healthcare [22, 23]. The country also faces challenges such as high suicide rates, high heavy drinking prevalence [24, 25], and an annual prevalence rate of 22% for psychological disorders [26]. A comprehensive overview of the Belgian psychiatric context can be found elsewhere [16].

### Procedure

Seven focus groups have been conducted. Focus groups were led by different investigators, which made it necessary to establish a topic guide to ensure consistency. In May 2022, two online brainstorming sessions were held with experts, with the aim of delineating the topics. Thirteen potential participants for the brainstorming sessions were invited by email. They were recruited based on their expertise in cultural psychiatry, such as staff members from the Red Cross working in Belgian refugee reception centers. Participants were asked to suggest names of other experts. In total, 15 experts took part in these preparatory online brainstorming sessions. The list of generated topics can be found in Table 1.

**Table 1.** Topic guide

Topic	Guiding questions
Cultural identity	How do we define ours and others’ culture and identity? How do cultural identity and patient rights vary across different groups?
Barriers to care	What prevents patients from diverse cultures from seeking care? What challenges do professionals face when providing cultural care?
Professional attitude	What core attitudes support inclusive, rights-based, culturally sensitive care? How open can institutions realistically be to cultural practices?
Welcoming and engaging practices	What makes an intake respectful and culturally responsive? What practical needs (e.g., diet, holidays, provider gender) should be considered in mental healthcare? How and when should family or context be involved?
Diagnostics	How can we distinguish between culture-bound expressions and psychopathology? How can cultural identity and context be assessed during diagnosis? What tools and disciplines support good cultural diagnostics?
Treatment	What builds trust and promotes effective treatment across cultures? What is the role of family, community, and culture in treatment? What should be avoided in cross-cultural therapeutic settings?
Recommendations	What support do professionals need to apply cultural guidelines? How can awareness, training, and structural conditions be improved?

### Participants

Three focus groups with professional caregivers, three with adult patients, and one with young adults in the transition age were conducted between March 9, 2023 and March 15, 2024, which is conform guidelines on the number of focus groups needed (*N* = 4–8) [27]. Next to the attending researcher(s), 5–10 participants attended the focus groups. All but one focus group were attended in person (Table 2). The researchers recruited the focus group participants from their own networks. The focus group, consisting of professional caregivers, was partly composed of participants from the online brainstorming sessions. Patients in the focus group on transitional psychiatry were recruited from two major psychiatric hospitals in Antwerp with a significant migrant patient population. The focus groups consisted of a mixture of first and second-generation migrants. The focus group on geriatric psychiatry consisted of members of the FAP geriatric psychiatry section.

**Table 2.** Focus group characteristics

No	Date	Participants	N	Researcher(s)	Data
1	February 9, 24	Professional caregivers: adult psychiatrists	5	KVDB	Live, audio recorded
2	March 1, 23	Professional caregivers: child and youth, adult and resident psychiatrists, psychologists	8	KVDB, MS	Online, audio recorded
3a 3b	March 9 and April 12, 23	Transition-age patients	5	KC	Live, notes, and feedback
4	January 16, 24	Patients	7	NA, KC, PN	Live, audio recorded
5	January 31, 24	Patients	7	NA, KC, PN	Live, audio recorded
6	March 15, 24	Patients	10	NA, PN	Live, audio recorded
7	May 4, 23	Professional caregivers: geriatric psychiatrists	10	MVDB	Live, notes, and feedback

Before starting the focus group, the objective of the study was explained, and participants were asked if they would feel comfortable with audio recording. No audio recording was made in the focus group with young adult patients in the transition age. Instead, the attending investigator took manual notes, which were afterwards shared with the attending participants for corrections. Manual notes were taken during the geriatric psychiatrists’ focus group due to a technical issue.

### Analysis

Data analysis software MAXQDA 24 [28] was used to code and analyze the focus group transcripts. Thematic analysis following the Braun and Clarke methodological framework guided the data handling: (1) data familiarization through transcription, (2) open coding of raw data, (3) axial coding to major data-driven inductive themes, (4) review of themes, and (5) interpretation of themes.

A researcher who was not involved in any of the focus groups (RW) analyzed all seven focus groups (steps 1–3) to avoid bias from prior knowledge or emotional connection. Data were manually coded line-by-line to identify meaningful units. Artificial intelligence and auto-coding features were not used. The codes captured not only predefined discussion topics but also emergent ideas raised by participants. Reflexivity was not only ensured by the independent position of the primary data analyst and by the use of reflexive memos throughout the coding process. It was also ensured by the initial use of descriptive coding (close to the data), which only after a first iteration was developed to more higher-level codes, and the use of a second reviewer. Author KC reviewed candidate themes and interpreted the thematic tree while rereading transcripts and coded extracts (steps 4–5). The process was iterative, with feedback loops between RW and KC, resolving discrepancies through discussion (steps 2–5). The thematic tree and representative quotes were then internally reviewed by the co-authors, who moderated a focus group to confirm researcher alignment with their focus group experiences. This internal review served as investigator triangulation rather than participant member-check. Results were not presented to participants because there was a substantial time gap between the focus groups and the data analysis, making it difficult to recontact the patients who participated. A member check with healthcare professionals might have thrown the analysis off balance.

## Results

**Figure 1.**
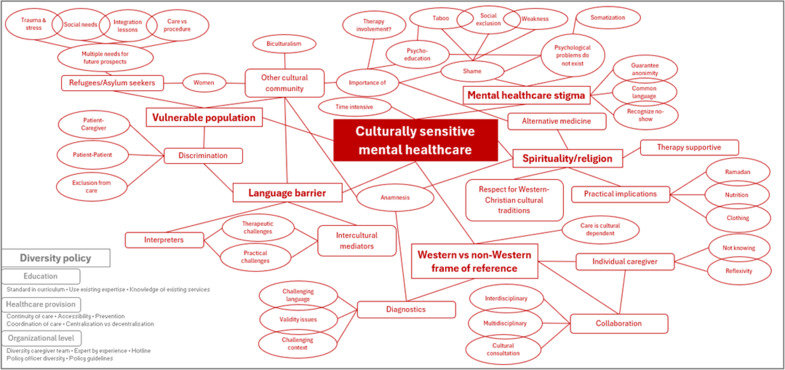
Thematic tree.

The thematic tree centers on “culturally sensitive mental healthcare” comprising five main themes (i.e., vulnerable population, language barrier, stigma, spirituality/religion, Western vs non-Western perspectives) and one overarching policy theme (i.e., diversity policy) (Figure 1). Each theme includes a number of problem statements followed by suggested solutions.

### Vulnerable population

#### Problem statement

All focus groups noted the vulnerability of patient populations with a migration background, with refugees and asylum seekers representing a particularly vulnerable population. Experiences of racism or discrimination in daily life may resurface in interactions with healthcare professionals and among patients themselves, sometimes linked to past traumatic events.

Black African people whom travelled across the Sahara countries do not want to hear any Arabic voices anymore, because it resembles a threat to them; it is a personal experience with a whole other meaning. – FG1 professionals

Cultural or religious misunderstandings of behavior can evoke feelings of unsafety in healthcare professionals, which again may result in discriminatory practices. Examples are speaking loudly in another language, more easily resorting to physically violent coping strategies, or showing frustration when unable to vocally express oneself.

Another specific challenge in migrant mental healthcare includes communication difficulties because of language barriers.

… I wanted to say something but my Dutch was not good. I named him a racist. He became angry and I did not understand why? – FG4 patients

The vulnerability associated with being part of a culture extends beyond the language barrier. Differences in gender roles can also add to the vulnerable position of female patients. Biculturalism (i.e., the state of belonging to or being influenced by multiple cultures) can be another stressful factor.

Wrapped between different cultures could lead to silencing of problems, out of fear to hurt or disappoint parents. – FG3 transition age patients

Family plays a central role in many cultures. Psychoeducation for families can help challenge certain cultural or religious views on mental health, for example, through multilingual brochures or informative family days. However, shame, anonymity concerns, and language barriers may limit participation.

#### Suggested solutions

Patients indicated that professionals should show respect and a willingness to listen, despite existing gaps in cultural understanding.

It happens a lot that patients accuse healthcare professionals of racism […] We may have a critical stance against it […] but healthcare professionals need to show some understanding, about what motivates the patient to react like that. – FG1 professionals

It has also been suggested that for refugees/asylum seekers, it is important to address both specific care needs and social needs, such as housing or access to integration lessons, to retain future perspectives as a human being. Extra attention should also be given to women, who are underrepresented.

90% of our refugees are men so extra attention should be given to female refugees, and more broadly female migrants. – FG2 professionals

To enhance family involvement, it has been suggested not to blame them and to provide psychoeducation also on the individual level. Although it should not be standard practice, involving family members in therapy (as stakeholders rather than interpreters) can support both patient and family, since differing views on (treating) mental health may conflict with the patient’s position as care seeker.

Psychological problems could cause loneliness, and finding love and support of friends and family could be lifesaving. – FG3 transition age patients

…the family often expects the patient to stay home, help with household tasks, and limit going out, as they are not viewed as marriage candidates. It is therefore important to look more broadly at what is expected of a daughter or son, and what is realistically achievable. – FG2 professionals

### Mental healthcare stigma

#### Problem statement

Perceptions of mental illness are heavily influenced by deep-seated cultural and religious beliefs. Mental health problems are often surrounded by shame, with affected individuals perceived as weak or personally responsible. Mental illness is generally regarded as more problematic than physical illness, while addiction and psychosis are considered less acceptable than depression.

Psychological diseases do not exist, psychological weaknesses do. […] In my country, a psychiatric diagnosis is a life sentence. You cannot go to work anymore. […]. It’s a stamp. – FG6 patients

Formal mental health facilities may be scarce in countries of origin. Mental illness may cause social exclusion as friends and community often turn their backs, while families typically continue to provide support and may push for alternative medicine.

In Africa, you are simply separated from society […] and every now and then you are subjected to shock treatment or some other pointless form of healing. It’s not really psychiatry as we know it here. – FG5 patients

In some cultures, there is a tendency to emphasize physical symptoms or to somatize psychological problems. Such somatic presentations are particularly relevant in cultural psychiatry, as they may be reinforced by limited access to care and by fears of not being taken seriously.

Sometimes, there are literally language issues when it comes to psychological problems. For some concepts, there is no right word in the culture or language of origin. – FG3 transition age patients

Shame can significantly hinder care provision, with anonymity becoming even more critical than in Western contexts. It may reduce participation in group therapy, limit willingness to involve family, and foster distrust toward third-party interpreters. Moreover, shame might negatively impact no-show rates.

#### Suggested solutions

Therefore, it has been suggested that healthcare professionals strive for a common language to foster trust with patients. Addressing physical symptoms may serve as a starting point for effective care. Healthcare professionals may need training on how mental illness and distress manifest across migrant communities. Greater awareness of culturally specific language, along with tailored explanations or adaptations, can be crucial for improving communication and care.

For instance, stress for an Afghan boy: stress is like everything is destroyed. While we are saying stress to everyday things. Same words but different meaning. – FG1 professionals

### Language barrier

#### Problem statement

There was a broad consensus that language and communication barriers often pose major obstacles to effective healthcare. The use of interpreters or intercultural mediators can help prevent frustration and escalation. While interpreters focus strictly on accurate translation, intercultural mediators go beyond words. Intercultural mediators may prevent misunderstandings, resolve conflicts, explain healthcare system customs, or help the professional understand the socio-cultural factors shaping a client’s distress.

The man from Palestine who lost close ones started to cite some Koran verses during the consultation. The Arabic interpreter would translate all Koran verses, while our Pashtun interpreter would simply say that it is getting religiously spelled out. The translation is being done differently. – FG1 professionals

However, practical challenges such as logistics, administration, and organizational cost considerations remain. Therapeutic challenges also arise: psychotherapy sessions with interpreters take longer, and patients may feel inhibited discussing sensitive topics in their presence. As translation is influenced by cultural frames of reference, interpreters should possess specific competencies for mental health care and should be professionals rather than family members or peers.

#### Suggested solutions

Therefore, it has been suggested to preferably use professional mediators and attune the provision of care to meet these practical and therapeutic challenges.

### Spirituality/religion

#### Problem statement

Perspectives on psychological problems vary between and within cultures, creating challenges in clinical practice. Not seldomly, family members of patients turn to spirituality/religion to seek explanations and solutions.

For instance, my family do look at psychosis differently than other people. They look at your spirit to treat something […] you are tormented by an evil spiritual power. – FG5 patients

Patients raised concerns about the limited accommodation of cultural practices in mental health services, particularly regarding nutrition and clothing. Moreover, residential care may conflict with Ramadan, while patients with a migration background generally reported no difficulties with Western-Christian cultural or religious traditions (e.g. Christmas, Eastern, Saint Nicholas). They embraced it and called it *cozy (FG5 patients).*

#### Suggested solutions

For patients for whom religion is important, healthcare professionals should acknowledge and consider religious beliefs during consultations, as these can significantly improve health outcomes, enhance patient satisfaction, and foster trust between patient and professional. Spiritual support such as interaction with religious leaders or allowing time and space to practice faith may contribute to patient well-being and indirectly support the healing process.

Take the example of a prison where imams come visit prisoners to offer them some redemption. They come to provide spiritual support, not to give them advice on their case. – FG4 patients

It was also suggested to evaluate the clinical feasibility of adapting care routines, day plans, and medication schedules to important traditions such as Ramadan.

I understand that the kitchen is not available at 4 am. But these are things that I would like to see changed. Then I wouldn’t had to make the decision to switch to day therapy. Now I felt like it was not possible at all. I cannot go to the mosque from here and pray and return in the middle of the night. – FG5 patients

It was emphasized that illness itself is a valid reason to abstain from fasting, with the option to compensate (bail out or catch up) later. Family and community members, however, could take a more skeptical stance toward mental illness due to the aforementioned stigma.

### Western vs non-Western frame of reference

#### Problem statement

Culturally appropriate care requires sensitivity to people’s cultural identity. Yet, Western-European healthcare professionals often approach patients through a Western lens. It is important to acknowledge that Western diagnostics may not be validated in non-Western populations. Diagnostic processes are further complicated by language and contextual challenges, underscoring the need for thorough cultural and religious anamnesis.

#### Suggested solutions

Adopting a “not-knowing” stance toward the patient’s frame of reference is crucial to culturally sensitive care. Reflexivity (i.e., the examination of one’s own beliefs, judgments, and practices) is a key skill competency in this regard.

You should at least acknowledge that you are stuck in a framework […] this is not the only way of evidence-based practice. That you are open-minded towards other frame of references and see how to build a bridge in-between. – FG2 professionals

The Cultural Formulation Interview (i.e., a structured interview exploring cultural aspects of mental health [29]) was also suggested as particularly useful. Inter- and multidisciplinary collaboration can help professionals reflect on their own culturally specific assumptions, while cultural consultation offers expert guidance to ensure culturally sensitive practice.

[…] a team composed of not only psychiatrists and psychologists, but also legal workers, political scientists, sociologists, creative therapists. This is important because many symptoms origins from trauma but sometimes also origins in the systems where they ended up. – FG2 professionals

### Diversity policy: The times they are a-changin’

#### Problem statement

Although openness toward cultural psychiatry has increased over time, its practical application and integration into mainstream clinical practice remain limited. This is due to a combination of conceptual difficulties, clinician-related factors (bias and training gaps), and systemic/organizational constraints.

#### Suggested solutions

Expertise concentrated in specialized centers should be incorporated into curricula and continuing education, supported by easily accessible platforms that inform professionals about available resources. The increasingly diverse student population also creates opportunities for peer learning. Importantly, cultural psychiatry training should extend beyond mental healthcare professionals to all disciplines.

Knowledge dissemination toward primary care providers and adolescent populations has been suggested as a possible prevention strategy to reduce pressure on specialized services. General practitioners’ offices and schools were highlighted as key settings for outreach, and the creation of peer group associations has been welcomed too. Continuity of care was emphasized through case management and outreach initiatives such as mobile teams.

At the policy and institutional level, a discussion is needed on the balance between centralized and decentralized services, and on whether cultural sensitivity should be embedded universally or delivered through specialized programs for specific issues.

Some specialization might be required, but we should acknowledge that you partly risk segregation. […] I said to be interested in cultural psychiatry and suddenly all patients were referred to me. Even patients with a simple depression but coming from South-Africa, while it has nothing to do with being from South-Africa. – FG2 professionals

It was suggested that organizations recruit more diverse healthcare teams, potentially through positive discrimination, and include individuals with lived experience or experts by experience. Appointing a dedicated diversity officer could further support the development of enforceable policy guidelines and initiatives such as hotlines.

## Discussion

This qualitative study explored the perspectives of patients and caregivers on culturally sensitive psychiatric care in Flanders, Belgium. We identified the central theme “culturally sensitive mental healthcare” with the following five main themes: vulnerable population, language barrier, mental healthcare stigma, spirituality/religion, Western vs non-Western frame of reference.

First, migrant groups were noted as especially vulnerable, particularly among adolescents and female asylum seekers. A recent review showed that adolescent immigrant populations may be at increased risk of various mental health problems [30]. Onyango et al. [31] further identified subgroups facing increased post-migration challenges, including pregnant women, forcibly displaced persons, and widows. This study also found that sociological strategies, such as providing culturally familiar nutrition, can improve mental health.

Second, as pointed out in earlier research [32], somatization emerged as both a manifestation of psychological distress and a potential entry point into care. A systematic review reported that migrants presenting with somatic symptoms experience greater psychological distress, higher perceived need for services, and more post-migration living difficulties and/or post-traumatic stress syndrome compared to those without somatization [33].

Third, professional interpreters and intercultural mediators were viewed as essential for effective communication and culturally attuned support, particularly during psychotherapy and crises. A recent study stressed the need for sufficient financial resources to guarantee equitable access to these services [34]. Clinical guidelines for working with interpreters have been published, covering key steps before, during, and after therapy sessions, as well as the use of translated assessment tools [35].

Fourth, spirituality and religion were recognized as central to how many patients with a migration background experience and cope with illness. These dimensions warrant attention in therapeutic encounters, requiring respect for patients’ explanatory models, meaning-making processes, and recovery hopes. A recent systematic review concluded that sensitive consideration of patients’ belief systems enhances culturally competent care, strengthening therapeutic relationships, and promoting shared decision-making [36].

Fifth, instruments have been developed to foster culturally specific mental healthcare. The Cultural Formulation Interview [29] can help collect information on patients’ experiences, help-seeking behavior, and treatment expectations. It has been found useful to improve communication and care planning [37], and it enhances diagnostics with notable shifts from depressive and psychotic disorders to trauma- and stressor-related disorders or absence of psychopathology [38]. At the organizational level, a self-assessment tool on five standards of equity in healthcare enables institutions to reflect on their current practices and whether they can do better [39].

These results are timely because the commitment of the Belgian mental healthcare system to cultural diversity remains weak [40], although the need for a paradigm shift and the call for culturally sensitive approaches to be embedded in health and social care systems are increasingly emphasized within Belgian expert communities [40–44] and in recent scholarly publications on concepts such as structural competency [7–9]. Initiatives are lacking to develop an inclusive mental healthcare that addresses the diversity and the needs of migrant and ethnic minority populations [40, 45]. For the case of Belgium, special attention may be drawn to people with a Moroccan or Turkish background [19]. This is in line with evidence showing that Belgium is among the worst performers when it comes to the education, employment, and housing of people with a migration background [46].

Drawing on focus group insights and aligned with published evidence, six recommendations are proposed to enhance culturally sensitive psychiatric care (Table 3).

**Table 3.** Six points of recommendation

1	**Every mental health professional should be aware of the additional psychological burden these patients face and take it into account in their practice.** Individuals with a migration background, particularly female refugees and adolescents, belong to the most vulnerable subgroups within mental health care. The reasons for this are multiple: immigration trauma, cultural and linguistic misunderstandings, the absence of a family network or the external pressure they may impose, socio-economic status, racism, discrimination, social exclusion, and stigma. The development of identity and personality in these patients must be actively supported by the social network and peers, but also by engaged caregivers. *Feasible action(s): (i) use instruments such as the Cultural Formulation Interview; (ii) develop outreach work plans to reach the most vulnerable.*
2	**Cultural psychiatry should occupy a central place in initial education and in continuing professional training of all mental health professionals.** In fact, all medical disciplines could benefit from such training. This includes the training and employment of more mental health professionals who share a similar background and/or native language proficiency as migrants. Patients with a migration background often express psychological distress through physical symptoms. Keeping this awareness of different symptom expression in mind can make the difference between stagnation and improvement for most patients. *Feasible action(s):* (i) *make use of the experiential knowledge within the increasingly diverse student population as an entry point to discuss the impact of culture on mental healthcare; (ii) scientific organizations may develop continuous training content on cultural psychiatry.*
3	**Training and availability of professional interpreters and intercultural mediators should be a top priority.** Many migrants do not speak the language of their new country. Without the proper use of language and accurate interpretation of cultural and religious backgrounds, it is impossible to be able to understand the patient’s inner world and behavior. However, the deployment of interpreters and mediators is not always appropriate and should be approached with due caution (see also recommendation five). *Feasible action(s): (i) develop an easy-to-use digital platform; (ii) assess local cultural psychiatry needs and tailor human resource allocation models.*
4	**Caregivers should investigate whether and how a patient’s psychiatric treatment program could benefit from complementary spiritual and religious support to foster the recovery process.** A basic attitude of respect and curiosity toward the patient’s religious and spiritual values strengthens trust and enhances the positive impact of treatment. *Feasible action(s): (i) build relationships with local religious or spiritual entities; (ii) provide psychoeducation to these entities; (iii) inquire whether and how healthcare organizational modalities can be aligned with cultural and religious matters; (iv) use instruments such as the organizational self-assessment tool MED-TF to learn and adapt practice if required.*
5	**Reflexivity** – the continuous examination of one’s own values, norms, and perspectives in relation to those of the patient – **and reciprocity** – the exchange between patient and caregiver - **are essential**. These attitudes are required to avoid potentially stigmatizing and discriminatory interactions within the treatment process, particularly when working with high-risk age groups. For example, group therapy, as well as the use of interpreters and mediators, may reinforce feelings of shame and taboo in patients with a migration background, and may have counterproductive effects in some cases. Paying attention to personal boundaries when discussing psychological complaints is the starting point. *Feasible action(s): (i) use cultural assessment tools such as the Cultural Formulation Interview; (ii) proactively discuss organizational and individual care plans with patients; (iii) discuss one’s own cultural beliefs in a group.*
6	**Establish several reference centers for cultural psychiatry.** Patients with severe and persistent symptoms should be able to be referred to reference centers. These reference centers could build interdisciplinary expertise and provide specialized advice to other caregivers to improve efficiency, as well as to support the dissemination of knowledge. Other institutions may evaluate, monitor, and improve their health equity standards with existing tools. *Feasible action(s): (i) use instruments such as the organizational self-assessment tool MED-TF to learn and adapt practice if required; (ii) determine the number of required specialized centers per region based on population demographics.*

### Strengths and limitations

A key strength of this study is the integration of opinions of both professionals and persons with lived experiences. Several limitations must also be acknowledged: (i) selection bias might have occurred as only data on hospitalized patients were collected, and all participants were recruited via professional networks; (ii) it was not possible to organize a focus group with family members with lived experience; (iii) main themes reoccurred across focus groups but not all topics were explored in equal depth, warranting further research. This points to thematic but not data saturation. For instance, participants noted that racism and discrimination from fellow patients toward individuals with a migration background are serious issues, occurring more often among patients than from healthcare providers. (iv) Although this was not the aim of our study, group dynamics or interactional elements that may have shaped how participants expressed their views were not analyzed. Future studies could incorporate a more detailed analysis of communication patterns to further contextualize the findings. (v) Participants did not have the opportunity to validate or refine our interpretations. Investigator triangulation and reflexive analysis were used to ensure the trustworthiness of the findings.

## Conclusion

Continued attention to the importance of cultural sensitivity in mental healthcare provision remains important, particularly in countries that are lagging behind. The recommendations provide a scientifically sound base to develop focused and effective mental health policies at governmental, organizational, and patient levels.

## Data Availability

Anonymized transcriptions (in Dutch) are available upon request with explicit justification (e.g., scientific purposes, verification).
